# A perforated anodised aluminium slide for improved specimen clearing and imaging for confocal laser scanning microscopy

**DOI:** 10.1186/s13104-018-3826-3

**Published:** 2018-10-10

**Authors:** Felix Simon Christian Quade, Beate Preitz, Nikola-Michael Prpic

**Affiliations:** 1 Georg-August-Universität Göttingen, Johann-Friedrich-Blumenbach-Institut für Zoologie und Anthropologie, Abteilung für Entwicklungsbiologie, Justus-von-Liebig-Weg 11, 37077 Göttingen, Germany; 2 Göttingen Center for Molecular Biosciences (GZMB), Ernst-Caspari-Haus, Justus-von-Liebig-Weg 11, 37077 Göttingen, Germany; 3 Justus-Liebig-Universität Gießen, Allgemeine Zoologie und Entwicklungsbiologie, Carl-Vogt-Haus, Heinrich-Buff-Ring 38, 35392 Gießen, Germany

**Keywords:** Confocal laser scanning microscopy, Tissue clearing, Imaging

## Abstract

**Objective:**

The bleaching, clearing and handling of tiny specimens with soft tissue and cuticular components for confocal laser scanning microscopy is difficult, because after cuticle bleaching and tissue clearing the specimens are virtually invisible. We have adjusted the design of the specimen container described by Smolla et al. (Arthropod Struct Dev 43:175–81, 2014) to handle tiny specimens.

**Results:**

We describe a perforated and anodised aluminium slide that was designed to hold the distal tips of the pedipalp appendages of the spider *Parasteatoda tepidariorum* during clearing, and that can then be used directly for confocal laser scanning microscopy. We believe that this slide design will be helpful for others who want to visualise specimens between 500 and 800 µm with confocal laser scanning microscopy.

## Introduction

Confocal laser scanning microscopy (CLSM) is a widely used visualisation technique in biology. Its major advantage is the capability to produce precise optical sections of a specimen, and these sections can be assembled into three dimensional reconstructions of the specimen using image processing software (e.g. Amira). The major limitation of CLSM is that the specimen has to be transparent to allow the laser to penetrate into the specimen. Thus, CLSM is mainly used for biological samples that are transparent or only slightly pigmented (e.g. embryos, many larval types). Many arthropods, however, have a strongly pigmented cuticle that prevents laser beams to enter the inside of the body. Smolla et al. [1] have devised a method to bleach the pigmented cuticle of insects and at the same time preserve and clear the soft tissue of the specimen. This method thus makes both the cuticle and the soft tissue fully transparent and allows the in situ documentation of internal soft tissue as well as cuticle with CLSM. We have transferred this method to the common house spider *Parasteatoda tepidariorum* and have applied it to the heavily pigmented pedipalp of the male. The protocol by Smolla et al. [1] worked very well, but we had difficulties with the handling of our tiny specimens (approx. 500–800 µm) after they had been fully bleached and cleared, because they were virtually invisible in the clearing medium and the 1 cm container described by Smolla et al. [1] proved too large for our specimens.

## Main text

**Fig. 1:**
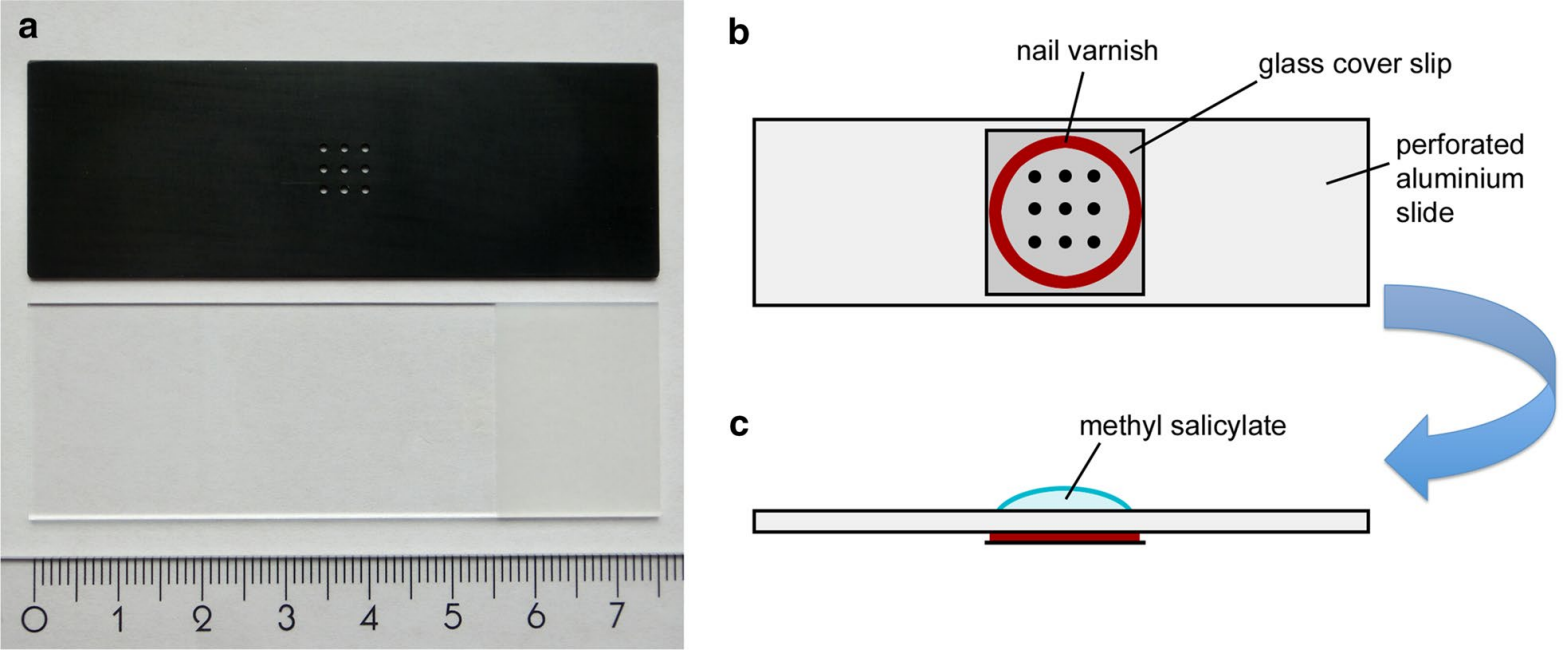
A perforated anodised aluminium slide for clearing and CLSM imaging of small specimens. **a** The anodised slide is shown at the top; note the holes that serve as containers for the specimens. A conventional glass microscope slide and a ruler (in cm) are shown below the slide for comparison. **b** Preparation of the aluminium slide for tissue clearing. A ring of nail varnish (denoted in red) and a cover slip are placed over the perforated area. **c** The slide is then turned upside down to place the specimens into the holes and to place a drop of methyl salicylate (denoted in blue) on the perforated area

We have devised a perforated aluminium slide that allows us to perform the clearing of several tiny specimens already on the slide that is also used for CLSM and therefore renders the further handling of the hardly visible specimens unnecessary. The slide was manufactured in our in-house precision mechanics workshop. It is made of aluminium alloy (DIN AlMgSi1) and has the dimensions of a regular glass microscope slide, approximately 75 mm × 25 mm × 0.5 mm (Fig. 1a). The slide was pre-cut from the metal sheet using a Festool Cs70 EB bench saw and was then milled to its final size using a milling machine (Deckel Maho DMU 50 T, CNC-controlled) equipped with an end-mill cutter of 4 mm diameter. In the centre of the aluminium slide we drilled 9 holes arranged in three rows. The diameter of the holes is 0.9 mm. The holes were drilled with the Deckel Maho DMU 50 T machine equipped with a twist drill bit (diameter 0.9 mm). These holes hold the specimens during clearing, but also allow the penetrance of the laser during imaging. In our experience in an untreated aluminium slide the laser light is reflected to a certain degree due to the small diameter of the holes. This laser reflection is problematic during imaging and reduces the image quality. We have therefore anodised the aluminium, so that the slide and, most importantly, the inside of the holes is lustreless and completely black. For anodising we used the Colinal technique (“**col**our **in** **al**uminium”; electrolytic colouring with Tin(II) sulfate) and the maximum colour depth EURAS C-35 (“black”). The process of electrolytic colouring has been performed in the central workshop for precision mechanics, Department of Physics, Göttingen. The black surface of the aluminium entirely prevented laser light reflection during imaging.

**Fig. 2:**
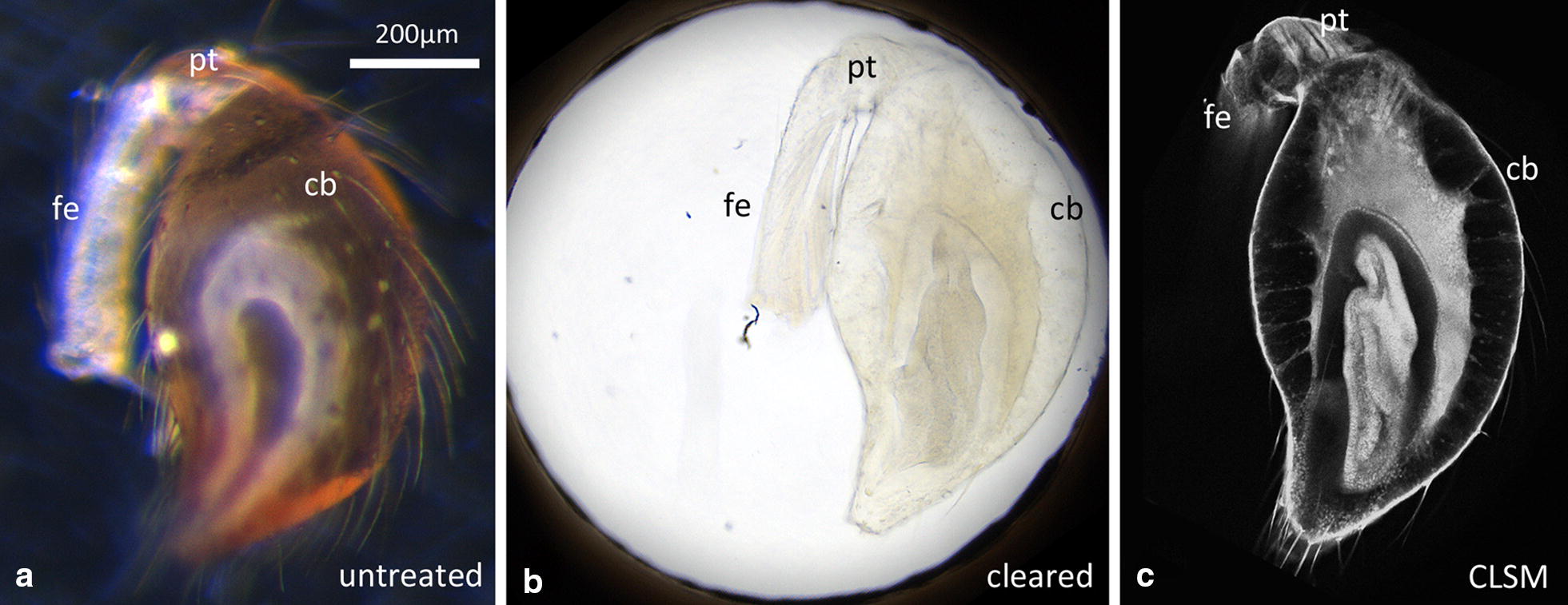
Specimen bleaching and CLSM imaging using the perforated anodised aluminium slide. **a** An untreated subadult pedipalp tip. The distal end comprises the segments femur (fe), patella (pt), and the club (cb). The club is surrounded by dark brown cuticle and contains internally (not visible) the primordium of the tibia, cymbium and the male copulatory organ (bulb). **b** A pedipalp tip fully cleared embedded in a hole of the perforated aluminium slide. The cuticle and the internal tissue are transparent. **c** CLSM image showing an optical section through the pedipalp tip. Internal anatomy of the club is well preserved (the primordium of tibia and cymbium surrounding the bulb organ). Scale bar: 200 µm (for all panels)

For tissue clearing, the slides were prepared as follows (Fig. 1b): around the square of 9 holes in the slide a ring of nail varnish was applied with a varnish brush. Then a square glass cover slip (18 mm × 18 mm × 0.17 mm) was placed on the nail varnish (lower the cover slip carefully, so that no nail varnish is pressed into the holes; the holes must remain entirely clean). After a few minutes the nail varnish has dried and fixes the cover slip tightly to the aluminium slide. The aluminium slide can then be turned upside down (Fig. 1c). The dehydrated specimens were then placed in the holes (one specimen per hole) and a sufficient drop of methyl salicylate was applied onto the specimens. The methyl salicylate should be added slowly and carefully to avoid washing out the specimens and to ensure that enough methyl salicylate enters the holes without air bubbles. After all holes were filled with methyl salicylate, a second glass cover slip was placed on top of the methyl salicylate in the perforated area, and excess methyl salicylate was carefully wiped away with an ethanol soaked, lint-free cloth. After an incubation time of approximately 60 min, the specimens were cleared (Fig. 2a, b) and the aluminium slide with the cleared specimens in the 9 holes was directly used for CLSM (Fig. 2c). Importantly, after the incubation time, the slide can also be turned upside down to scan the specimens from the other side. This facilitates the scanning of objects that are too deep to be scanned from one side only.

## Limitations

The basis for our perforated anodised aluminium slide is the specimen chamber described by Smolla et al. [1], which proved to be too large for our purpose of clearing the tiny pedipalp tips of *P. tepidariorum*. The slide described in this note is therefore specially designed for tiny specimens, of about 500–800 µm. Larger specimens are better handled in the specimen chamber as described by Smolla et al. [1]. We have no experience with specimens smaller than 500 µm, but we believe that the diameter of the holes can be made less than 0.9 mm to suit smaller specimens, if the inside of the holes is properly anodised to prevent laser light reflections.
